# The Oldest Bryophyte Herbarium Specimens from Central Europe, Collected by M. E. Boretius in 1717: Taxonomy, Nomenclature, Datation and Ethnopharmacology

**DOI:** 10.3390/plants13030349

**Published:** 2024-01-24

**Authors:** Jacek Drobnik, Adam Stebel, Maja Graniszewska

**Affiliations:** 1 Department of Pharmaceutical Botany, Faculty of Pharmaceutical Sciences in Sosnowiec, Medical University of Silesia in Katowice, ul. Ostrogórska 30, 41-200 Sosnowiec, Poland; astebel@sum.edu.pl; 2 The WA Herbarium, Faculty of Biology, University of Warsaw, ul. Żwirki i Wigury 101, 02-089 Warsaw, Poland; m.graniszewska@uw.edu.pl

**Keywords:** herbarium, bryophytes, 18th century, Poland, medicinal plants

## Abstract

The WA Herbarium at the University of Warsaw houses a collection of plants created in 1717 by Matthew Ernest Boretius. They were gathered in former East Prussia, near Angerburg, now Węgorzewo (Poland). It is the oldest plant collection from this part of Europe. Boretius compiled the herbarium as a collection of all the surrounding plants, but their folk names (Polish and German) recorded in the herbarium confirm the ethnobiological or ethnopharmaceutical importance of some species. We identified bryophyte species and checked the accuracy of their original identifications recorded in the herbarium. We provided their Latin (scientific, pre-Linnaean) nomenclature together with German and Polish vernacular names. We contextualised this information within the history of the medicinal use of bryophytes around 1717, when the plant collection was created. We also investigated whether the specimens could have come from Northeastern Poland. Mosses and liverworts from the herbarium were identified nomenclaturally (by means of their original scientific polynomial names written on herbarium sheets) and taxonomically. The herbarium holds two species and one subspecies of liverwort and 27 species and one variety of moss. The accuracy of the original identifications was assessed, with a particular focus on the species considered medicinal at the time. We found that bryophytes were poorly known in the time of Boretius, which was the last period in bryology before the introduction of magnifying devices into this science (this crucial step was made by Dillenius in 1741). The vernacular names used in the herbarium were recorded for *Marchantia polymorpha* and *Polytrichum commune*—the only two species with confirmed medicinal use by the year 1717.

## 1. Introduction

Historical plant collections not only inform us about their former occurrence and diversity. They also reveal the old botanical nomenclature, which is not always published. In addition, the annotations on the herbarium sheets, the arrangement of the sheets in the herbarium, and the wider context of the origin of the collection and its creator can indirectly provide new ethno pharmacological and ethno botanical knowledge. 

**The collection.** The WA Herbarium of the Faculty of Biology at Warsaw University houses a historical collection of plants that come from the former Prussian town of Königsberg (Polish: Królewiec, now Kaliningrad in the Russian exclave). The authorship of the collection in question has long been disputed. Until now, it has been attributed to either Helwing or Boretius, or both, and the date of creation is uncertain. We call it here the WA copy.

**Matthias Ernst Boretius**, a Prussian botanist and physician, was born in 1694 in Lötzen (now Giżycko, Poland) and died on 4 October 1738 in Königsberg (now Kaliningrad, Russia). He was a professor of medicine at the University of Königsberg, a city physician since 1728 [1], and a court physician to the Prussian king Friedrich Wilhelm I [2], but only since 1738 [1]. In his study of botany, Boretius was a pupil of Georg Andreas Helwing (1666–1748), a prominent Prussian naturalist. Boretius was the author of four botanical–medical works, including his doctoral dissertation [1]. He became Helwing’s son-in-law [3]. 

A Prussian historian, Georg Christoph Pisanski (1725–1790), who was a grandson of Helwing, claimed that the herbarium had been created by Boretius under Helwing’s supervision [4] (p. 549).

**Copies of Boretius’ herbaria**. In 1886, Caspary [5] communicated his discovery in an old Prussian newspaper *Wochentliche Königsbergische Frag- und Anzeigungs-Nachrichten*. The library resources of this newspaper from the 1730s are now lost, so we must rely on Caspary’s account. We reproduce his report in full:

“Georg Andreas Helwing became assistant to his father, who was a clergyman in Angerburg [today Polish: Węgorzewo], in 1691, became provost in Angerburg in 1725 and died in 1748. From spring 1717 onwards, the medical student Mathias Ernst Boretius, later *professor ordinarius tertius* at the medical faculty in Königsberg, who had to study botany, died in 1738, stayed with him for a longer period in order to acquire botanical knowledge from Helwing, the author of the [books] *Flora quasimodogenita* and *Florae campana*, and an excellent plant expert. In 1717, Boretius made herbaria under Helwing’s supervision, they were distributed to various prominent people. In the *Wochentliche Königsbergische Frag- und Anzeigungs-Nachrichten* from the year 1737, volume 27, a journal that gave information about all things of practical life […] but also [published] treatises of the most important scholars of Königsberg University (e.g., also by Immanuel Kant)—in this journal Boretius says in a treatise [entitled]: *Von Nana Oder Ananas und deren Frucht* (‘Of *Nana* or Pineapple and its Fruit’): «After them (i.e., the Prussian botanists Wigand, Wolff, Mentzel, Loesel, Gottsched), the highly experienced and indefatigable M. Georg Andreas Helwing, the current provost in Angerburg, has searched out almost as much in the Prussian forests, shrubs and fields, as the *Herbaria viva* produced under his supervision 20 years ago, one of which has the honour of being preserved in the Royal Library in Dresden of His Majesty the King of Poland for more than some time.» This [Boretius’] account was published in 1737; the herbaria were therefore made by Boretius in 1717. Unfortunately, what the then King of Poland and Saxony received was burnt during the revolution in Dresden in 1848, or, as it is also called, came to Petersburg. One was given to the city secretary of Danzig: Jacob Theodor Klein. Three are in Königsberg, one in the Royal library, one in the Municipal [Library] and one in the Royal Botanical Garden. The last one was owned by Carl August Hagen, the author of [books]: *Chloris Bor[ussica] und Preussens Pflanzen* and was recently given to him by Hagen’s grandson. It [i.e., Boretius’ herbarium] consists of 5 thick volumes of writing paper bound in pigskin *in folio*, in which the plants are pasted, given the long names they had in Helwing’s times, and mostly also by C. G. Hagen’s hand with Linnean [names]. The latter was owned by Carl August Hagen and was recently donated to the Royal Botanical Garden by Hagen’s grandson, the present court pharmacist Hagen.”

[5]

This discovery was soon summarised by Conwentz [6]. The latter author described the copy of Boretius’ herbarium, which was owned by the aforementioned Jacob Theodor Klein (1685–1759), as part of Klein’s natural history cabinet. Klein’s collection was bought by Friedrich, margrave of Brandenburg-Bayreuth, and moved to the old castle in Bayreuth. This copy was described by [6] as follows:

“The herbarium consists of 5 thick leather-bound folio volumes, the covers of which are decorated with coloured plant illustrations. The dried specimens are glued to strong writing paper and are generally well preserved. The Latin designation is cumbersome, as it was in use before the introduction of Linnean nomenclature.”

[6]

The author [6] mentioned five ornately bound volumes, and, in his article, he also reprinted the title page of the first volume, which might have been added whilst the volumes were being specially bound. The title page of Klein’s copy reads, in Latin, *Herbarium Prussicum ad Methodum illustris Tournefortii XXII Classibus absolutum et V Tomis adornatum, Studio ac Opera Viri plurimum reverendi Domini M. Georg. Andr. Helwingii, praepositi Angerburgensis et Societ. Scient. Berol. membri in gratiam mei I. Th. K. anno MDCCXXV. Accesserunt Index trilinguis ad Calcem Tomi V.ti, et separatum MSCtum Dni Helwing: Tournefortus Prussicus*. Thus, we can see that a new title, *Prussian herbarium*, was added, and the new year, 1725, appeared, probably on the occasion of the rebinding of the books at or after the donation to Klein. Moreover, the whole herbarium was accompanied by a separate handwritten (“*M*[anu]*SC*[rip]*tum*”) brochure by Helwing entitled *Tournefortius Prussicus*. The existence of such a supplementary work was confirmed by [4] (p. 549). 

Klein’s copy, which was housed at the University of Erlangen until Conwentz’s time and was transferred from Erlangen to Danzig (Polish: Gdańsk) before 1888 on the initiative of Conwentz himself [6], is now lost [7]. 

The WA copy has no title page in any volume, and it has simple bindings, so we should consider its condition and form to be original. 

**Authorship.** The two overlooked accounts [5,6] from the years 1888–89 resolve our earlier doubts about the authorship. We should also rely on the oldest bibliographical entry [8] from the catalogue of the Königsberg Municipal Library, which matches this collection with Boretius. There is also another herbarium in the National Library in Warsaw, which is undoubtedly the work of Helwing: the handwriting in this Helwing herbarium differs considerably from the handwriting in the WA copy [9]; for samples, see [10].

**Notes on the contents of the copies of Boretius’ herbaria.** Caspary [5] dealt with the first appearance of *Senecio vernalis* L. in East Prussia. He found a specimen of this species in the copy of Boretius’ herbarium kept at the Royal Botanical Garden in Königsberg. He wrote that the plant was included in vol. 4 on p. 66. The same species in the same volume number, and on a page with the same number, is included in the WA copy of Boretius’ herbarium. The page with *S. vernalis* in the WA copy is signed in Latin in the same way (polynomial name), but the plant is structured differently (Caspary described the structure of the plant in detail). The author of [6] listed more peculiarities from Klein’s copy (but without page numbers):

- *Pedicularis candida florubus candidis*, which is probably a hybrid of *Odontites vulgaris* Moench. A specimen with the same polynomial exists in the WA copy (vol. 1, p. 183).
- Two imperfect forms of *Geum rivale* named *Caryophyllata foliis Hederae terrestris* and *Caryophyllata prolifera flosculis elegantissimis*. Both these forms are also present in the WA copy in vol. 2, p. 238 and p. 236, respectively.
- One imperfect form of *Thymus pulegioides*, named *Serpillum capitulo tecto seu abortivum*. Such a form and the name are also present in the WA copy in vol. 1, p. 236.
- One imperfect *Plantago major*, named *Plantago latifolia rosea, floribus quasi in spicam dispositis*, which is also present in the WA copy in vol. 1, p. 83.
- One malformed specimen of *Veronica anagallis-aquatica* L. with galls in inflor escence, named *Veronica pilulas ferens seu Anagallis pilulifera Mentzelii*. It is also present in the WA copy in vol. 1, p. 151.

The names of all these unusual plant forms listed by [6] are identical to those in the WA copy. Based on these coincidences, including the absolute rarities and their identical names, we can now claim that the contents of all copies of Boretius’ herbarium were identical and probably created at the same time. 

**The WA copy of Boretius’ herbarium** was originally kept in the former public municipal library (German: *Öffentliche Stadtbibliothek in Königsberg*). This historical object was catalogued in Latin as *Matth. Ern. Boretii Herbarium vivum, Plantarum et Florum in Porussia nascentium Methodo Tournefortiana, in Classes divisum; adscriptis Nominibus Plantarum Latinis, Germanicis, Polonicis, cum Indice. Vol. V.* Translation: “Matthew Ernest Boretius’ living herbarium of plants and flowers growing in Prussia, divided into classes according to Tournefort’s system, supplemented with Latin, German and Polish plant names, with an index, five volumes” [8] (p. 66). After the turbulent events of the Second World War, four volumes survived in the WA herbarium, while volume no. 3 is missing. Each volume bears the stamp of the Königsberg Municipal Library [7]. The original library reference numbers are given on the inside back cover of each volume. They range from “422.1” to “422.5”. The reference number of this object in the WA inventory is WA-KH-13.1. This set of volumes will be referred to here as “the WA copy”.

**Datation.** According to Boretius himself, he created his herbaria in 1717 [5]. Some printed botanical works are cited in the volumes, notably the *Flora quasimodogenita* [11]. The collection could also be expanded and annotated with plant nomenclature and bibliographical references after 1717. See the *Discussion* for more datation-related facts. 

**Arrangement of plants.** Boretius’ herbarium is arranged according to the system of Tournefort [12], established in 1700. He divided the plants into 22 classes according to the structure of the flowers. Class XVI included organisms that did not produce flowers: ferns and some lichens. Class XVII consisted of organisms that produced “neither flowers nor fruits”: algae, fungi, bryophytes, Lycopodiopsida, and some other lichens [9], or at least plants that were mistakenly recognised as such.

**Bryophytes.** In the fifth volume of Boretius’ herbarium, mosses (*Bryophyta*) are members of the class named in Latin *Classis XVII exhibens herbas et suffrutices quorum flores et fructus vulgo desiderantur*. Translation: “Class 17 showing herbs and prostrate shrubs whose flowers and fruits are generally desired”. Liverworts *Marchantiophyta* are members of the class defined in Latin as *Classis XVI exhibens herbas et suffrutices qui floribus carent et semine donatur*—“Class 16 showing herbs and prostrate shrubs that are devoid of flowers and are endowed with seed”—and were placed in the fourth volume.

## 2. Aims of the Work

We identified bryophyte species included in the WA copy of Boretius’ herbarium and ascertained whether their original identifications made by Helwing for Boretius or by Boretius himself were correct in view of modern taxonomy. We determined their Latin (scientific, pre-Linnaean) nomenclature as well as German and Polish names. We present these data against the history of the medicinal usage of bryophytes in past centuries. We also checked whether the specimens represent the local flora of Northeastern Poland.

## 3. Materials and Methods

Bryophyte specimens were identified by two authors in two independent ways: A.S. identified the species taxonomically. J.D. read the original polynomials handwritten in the herbarium and tracked their synonymy to the present day. These results were then compared to assess the accuracy of the identifications (i.e., Latin polynomial species names) written in the herbarium.

### 3.1. Taxonomy and Floristics

Bryophytes were identified using a magnifying glass (Nikon SMZ1500, Nikon, Tokyo, Japan). Due to the historic nature of the herbarium, no specimens were sampled for microscopic examination. However, most of the bryophytes in the herbarium are common, large, easily identifiable species. The names of the identified species correspond to the latest revision in the “WFO Plant List” [13] and the sources mentioned therein, as well as [14]. 

Based on historical information (including Boretius’ biography), we assumed that the specimens had been collected near the town of Węgorzewo (formerly Angerburg) in Northeastern Poland. This allowed us to treat the bryophytes in the herbarium as representatives of the local flora at the beginning of the 18th century. Thus, we compared the occurrence of the identified species in the wild with the results of recent floristic research around Węgorzewo, published by [15,16,17].

### 3.2. Historical Naming of the Species

The interpretation of the names of bryophyte species recorded in the herbarium was the second independent method of our study. The names inscribed next to the specimens were searched for in the botanical literature published since the 16th century, and the successive synonyms found for these names, given by successive botanists, formed a chain that eventually led us to the modern accepted binomial. In almost all cases, the chain of synonyms led to the book by Dillenius (J. J. Dillen) [18], a key work for the pre-Linnaean nomenclature of bryophytes. Dillenius’ names were then traced as synonyms of the binominal names given by Hedwig [19], which is the nominative source for mosses. Liverworts were traced back to Linnaeus’ work, which is the nominative source for this group of bryophytes. The currently accepted binomials were found in [14]. This allowed us to automatically detect polynomial basonyms that were established in Boretius’ herbarium and subsequently published (or not published) by Helwing [11,20].

### 3.3. Historical Medicinal Usage

Johannes Boretius, as a physician, was familiar with plants, which, at the time, were the most important source of medicine. Bryophyte species known as medicinal plants in 1717 (based on our earlier research [21,22] and accounts cited therein) were indicated. We paid special attention to their German and Polish vernacular names included in the herbarium, recognising that names in national languages may provide evidence of the actual use of the species in question as medicinal herbs or more generally as economic plants.

## 4. Results

Below, we present the contents of each herbarium page where true bryophytes are found. We include transcriptions of the original handwritten names, their proper citations, the chain of synonymous polynomials, and its result as the accepted binomial (sometimes, more than one species was finally identified). Below each original name and its nomenclature, we give the independent taxonomical identification of each specimen, with the species name given in **bold**.

A total of two species and one subspecies of liverwort and 27 species and one variety of bryophyte were identified from the plant specimens collected in the herbarium.

### 4.1. Volume 5, Page 1

Page 1 holds one specimen with one Lat. name: *Muscus capillaceus major stellatus, floribus in apice coccineis expansis. Boerh.* There are two close published polynomials:

- *Muscus capillaris floribus in apice coccineis expansis Buxbaum Cent. 1. T. LVII f. 2.* It was published by [23] (p. 42). This Buxbaum polynomial was cited by Haller, who synonymised it with *Polytrichum vulgare et majus capsula quadriangulari Dill. Syn. III p. 90 n. 1.* [24] (p. 106). Dillenius [18] (p. 423) synonymised it with his *Polytrichum quadrangulare vulgare, Juccae foliis serratis* (p. 420), which, according to [19] (p. 88), is the Linnean *Polytrichum commune* L. [25] (p. 1573). However, Buxbaum’s name was published after 1717, the year of creation of the herbarium, and might not have been in use before 1728.
- *Muscus capillaris floribus in apice coccineis expansis Ind. Alt. 1. p. 21.* The abbreviation Ind. Alt. refers to the work by Boerhaave [26] (p. 21). Haller synonymised it with his own *Polytrichum montanum et minus, capsula quadrangulari* [24] (p. 107). Haller’s polynomial was later synonymised by Dillenius [18] (p. 425) with his *Polytrichum quadrangulare juniperi foliis brevioribus et rigidioribus* [18] (p. 424), which was eventually assigned by [19] (p. 90) with the Linnean species *Polytrichum commune* L. var. β [25] (p. 1573), today *Polytrichum juniperinum* Hedw. [19] (p. 89).

**Identification:** the specimens represent ***Polytrichum commune* Hedw.** (right side) with an admixture of ***Polytrichum* cf. *strictum* Menzies *ex* Brid. [=*Polytrichum juniperinum* Hedw. subsp. *strictum* (Menzies *ex* Brid.) Nyl. & Säl.]** (left side). They grow on peat bogs and in marshy forests and have been recently found near Węgorzewo [15,16]. 

### 4.2. Volume 5, Page 2

Page 2 holds one specimen with five names:

Lat.: *Muscus capillaceus major, pediculo et capitulo crassioribus*. It was classified by Dillenius [18] (p. 22) as a synonym for his *Polytrichum quadrangulare vulgare, Juccae foliis serratis* (p. 420), and by [19] (p. 88) as a synonym of *Polytrichum commune* L. from [25] (p. 1573). 

Lat.: *Adianthum aureum Fl. qsm*. This citation indicates the *Flora quasimodogenita* by Helwing [11]. On p. 23 of this book, it is a synonym for *Adianthum aureum Tabern.* = *Polytrichum aureum majus C. B. Pin. 356*. The latter polynomial by Bauhin [27] (p. 356) was synonymised by Linnaeus [28] (p. 1109) with his *Polytrichum commune* L., today *P. commune* Hedw. 

Lat.: *Polytrichum majus*. This name, attributed to Tragus, is used in Bauhin [27] (p. 356) and is another synonym of the taxon identified above. 

Germ.: *Groß gülden Wieder-Todt* and *Frauen oder Venus-Haar*. These two German names exist also in the flora by Helwing [11] (p. 23). They can be rendered as “great gold death-again” and “Feminine or Venus-hair”.

Pol.: *Matki Bozey Włoski*, literally “God’s Mother’s hair”, i.e., “Our Lady’s hair”. 

**Figure 1:**
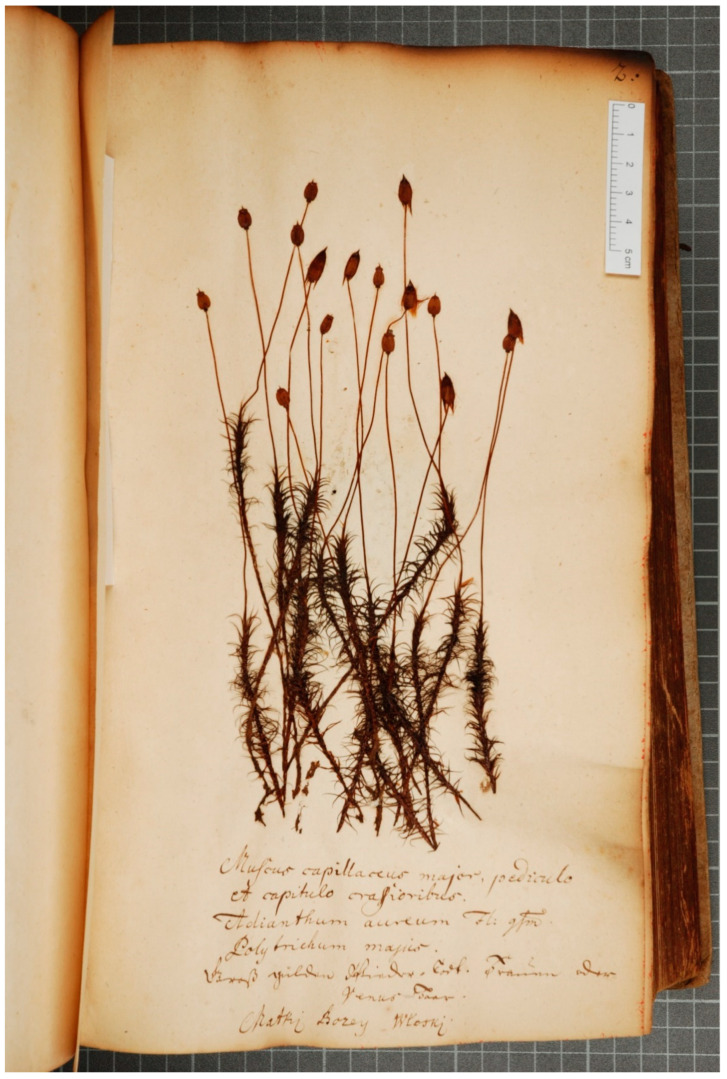
*Polytrichum commune* Hedw. in the WA copy of Boretius’ herbarium from 1717. Photograph by M. Graniszewska. Transcription: *Muscus capillaceus major, pediculo et capitulo crassioribus. Adianthum aureum Fl. qsm*. *Polytrichum majus*. *Groß gülden Wieder-Todt*. *Frauen oder Venus-Haar*. *Matki Bozey Włoski* (see Section 4.2 for translation and interpretation).

**Identification:** the specimens represent ***Polytrichum commune* Hedw.** again. See Figure 1.

### 4.3. Volume 5, Page 3

Page 3 holds one specimen with one Lat. name: *Muscus capillaceus major capitulo et pediculo tenuioribus*. This polynomial can be cited after several authors, mainly Tournefort [12] (p. 551) and Vaillant [29] (p. 82). It was synonymised by Dillenius [18] (p. 359) with his *Bryum reclinatum, foliis falcatis, scoparum effigie* (p. 357). Moreover, [19] (p. 126) named it *Dicranum scoparium* Hedw. 

**Identification:** the specimens represent ***Dicranum scoparium* Hedw.**, occurring mainly on the forest floor in coniferous forests, as well as on rotten wood, the bark of trees, and rocks. It still grows near Węgorzewo [15,16].

### 4.4. Volume 5, Page 4

Page 4 holds one specimen with three names: 

Lat.: *Muscus capillaceus minor folio breviore, capitulo nutante*. In [18] (p. 409), this polynomial is authorised by *Tourn. Hist. Pl. Par. p. 498*, i.e., Tournefort [30] (p. 498), and by *I. R. H. p. 551* [12] (p. 551). It was synonymised by Dillenius [18] (p. 407) with his *Bryum bulbiforme aureum, calyptra quadrangulari, capsulis piriformibus nutantibus*. Meanwhile, [19] (p. 172) named it *Funaria hygrometrica* Hedw. 

Lat.: *Adianthum aureum minus Loes*. This polynomial is present as *Adianthum aureum minus Tab. lib. 2 fol. 476* in [31] (p. 6). It was listed by Dillenius [18] (p. 480) as *Adiantum aureum minus* (with different and various authorship citations) as synonyms for his *Bryum bulbiforme aureum, calyptra quadrangulari, capsulis piriformibus nutantibus*. It is again *Funaria hygrometrica* Hedw. [19] (p. 172). 

Germ.: *Klein Wieder-Todt oder Venus-Haar*. Meaning: “small death-again or [small] Venus-hair”.

**Identification:** the specimens form a mixture of ***Ceratodon purpureus* (Hedw.) Brid.** (mainly from the left side) and ***Pohlia nutans* (Hedw.) Lindb.** (mainly from the right side). *C. purpureus* is one of the commonest Polish mosses; it grows in various habitats in forests and non-forest vegetation. *P. nutans* is a common moss, occurring mainly in forests on soil, rotting wood, and rocks, also in patches of psammophilous grassland. Currently, they are among the most common mosses in the bryoflora of the Węgorzewo region [15,16].

### 4.5. Volume 5, Page 5

Page 5 holds one specimen with three names: 

Lat.: *Muscus stellaris roseus C. B.* This polynomial should be cited after Bauhin [27] (p. 361). In Dillenius [18] (p. 412), it was made a synonym for his *Bryum dendroides polycephalon, Phyllitidis folio undulato pellucido, capsulis ovatis pendulis*, which later became *Mnium roseum* Hedw. [19] (p. 194), now *Rhodobryum roseum* (Hedw.) Limpr. [22,32].

Lat.: *Muscus erectus foliis in orbem sparsis Loeselii*. This polynomial is present in [31] (p. 168). It was classified by Dillenius [18] (p. 412) as a synonym for his *Bryum stellare roseum majus, capitulis ovatis pendulis* (p. 411). Both these names are missing in [19]. They are identifiable as *Rh. roseum*

**Identification:** the specimen is a mixture of ***Plagiomnium* cf. *affine* (Blandow *ex* Funck) T.J.Kop., *Eurhynchium angustirete* (Broth.) T.J.Kop.** and ***Rhodobryum roseum* (Hedw.) Limpr.** These three species grow on the ground in forests. They are still present in the vicinity of Węgorzewo [15,16,17].

### 4.6. Volume 5, Page 6

Page 6 holds two specimens. The top specimen has four names:

Lat.: *Muscus squamosus major s: vulgaris.* It should be cited after Tournefort [12] (vol. 1, p. 553) and Vaillant [29] (p. 137). Later, Dillenius [18] (p. 294) made it a synonym for his *Hypnum vulgare triangulum maximum et pallidum* (p. 293). For this name, Hedwig [19] (p. 256) applied his binomial *Hypnum triquetrum* Hedw., today *Hylocomiadelphus triquetrus* (Hedw.) Ochyra & Stebel. 

Lat.: *Muscus terrestris vulgaris Loes.* A similar polynomial existed, *Muscus terrestris vulgaris Lob. ic. pag. 151*, i.e., from [33] (p. 151), and it was also listed in [34] (p. 49). It is identifiable as *Rhytidiadelphus loreus* (Hedw.) Warnst.; for a discussion, see [22] (pp. 394–395). 

Germ.: *Brunnen Erd-Moß*, literally: “brown ground-moss”.

Pol.: *Mech*, literally: “moss”. 

**Identification:** the specimen is ***Cirriphyllum piliferum* (Hedw.) Grout**, which is a moss associated with wet forests and meadows, still present in the flora of the vicinity of Węgorzewo [15,16].

The bottom specimen has one Lat. name: *Muscus squamosus ramosus tenuior capitulis Adianthi aurei Raij.* This polynomial seems confused, and the closest correct names are the following:

- *Muscus terrestris vulgaris minor adianti aurei capitulis* in [35] (p. 625), which Dillenius [18] (p. 196) made a synonym for his *Hypnum dentatum vulgatissimum operculis obtusis* (p. 295). In [19] (p. 296), the latter name is a synonym for Hedwig’s species *Hypnum rutabulum* Hedw., today *Brachythecium rutabulum* (Hedw.) Schimp.
- *Muscus squamosus ramosus tenuior, capitulis incurvis* by [30] (p. 502) and [29] (p. 138), which is placed in [18] (p. 327) as a synonym for his *Hypnum velutinum, capsulis ovatis cernuis* (p. 326). It was named *Hypnum albicans* Hedw. and is today *Brachythecium albicans* (Hedw.) Schimp.; however, *materia medica* writers identified it as *H. velutinum* Hedw., today *Brachytheciastrum velutinum* (Hedw.) Ignatov & Huttunen.

**Identification:** the specimen represents ***Eurhynchium angustirete* (Broth.) T.J.Kop.**, a species moss associated with forest soil, still present in the bryoflora near Węgorzewo [15,16].

### 4.7. Volume 5, Page 8

Page 8 holds one specimen with four names:

Lat.: *Muscus squamosus major foliis angustioribus acutissimis. Tourn.* This polynomial should be cited after Tournefort [12] (vol. 1, p. 553), in which place it is also synonymised with *Muscus montanus* [36] (p. 809). Dillenius [18] (p. 305) named it *Hypnum loreum montanum, capsulis subrotundis*; then, [19] (p. 294) established a binomial, *Hypnum loreum* Hedw., today *Rhytidiadelphus loreus* (Hedw.) Warnst. 

Lat.: *Muscus terrestris repens Lycopodii ferme facie Dod.* This polynomial can be sufficiently cited after Ray [37] (p. 337) and so it is in [18] (p. 272). The latter author named it *Hypnum pennatum undulatum, Lycopodii instar sparsum* [18] (p. 271). It was named *Hypnum undulatum* Hedw. [19] (p. 242), today *Plagiothecium undulatum* (Hedw.) Schimp. [=*Buckiella undulata* (Hedw.) Ireland].

Lat.: *Muscus denticulato similis C. B.*, (originally: *Musco denticulato similis*) should be cited after [27] (p. 360). Dillenius [18] (p. 305) made it a synonym for *Hypnum loreum montanum, capsulis subrotundis*. The latter became *Hypnum loreum* Hedw. [19] (p. 294), today *Rhytidiadelphus loreus* again.

Germ.: *Berg-Moß.*, i.e., “montane moss”.

**Identification:** the specimens represent ***Climacium dendroides* (Hedw.) F.Weber & D.Mohr**. This moss still grows in this area in wet meadows, wet forests, and thickets [15,16].

### 4.8. Volume 5, Page 9

There are two specimens. The top one is named in Lat. *Muscus denticulatus major pulcher, parvus, repens J. B.* In the cited source [38] (p. 765), this species is named *Muscus pulcher parvus repens*. The latter polynomial was synonymised by Dillenius [18] (p. 466) with his *Lycopodioides denticulatum pulchrum repens, spicis pediculis infidentibus*. It is absent in Hedwig’s [19] work because this name indicates *Selaginella helvetica* (L.) Spring, not a bryophyte. 

**Identification:** the top specimen is ***Neckera pennata* Hedw.**, nowadays an exceedingly rare epiphytic species near Węgorzewo, which was found only on well-preserved patches of old forests [16].

The bottom specimen is named in Lat. *Muscus denticulatus minor C. B. confer. Tourn. p. 556*. The polynomial *Muscus denticulatus minor* is here cited correctly after [27] (p. 360) and [12] (vol. 1, p. 556). By Dillenius [18] (p. 463), it was made a synonym for his *Lycopodioides imbricatum repens*. The latter in [25] (p. 1569) is *Lycopodium denticulatum* L., today *Selaginella denticulata* (L.) Spring, which is not a moss.

**Identification:** the bottom specimen is also ***Neckera pennata* Hedw.**, but with sporophytes.

### 4.9. Volume 5, Page 13

Page 13 holds one specimen with one Lat. name: *Muscus densis foliolis Juniperinis in cespitem congestis Boerh.* This polynomial is absent in printed books; the closest variant is *Muscus, densis foliolis juniperinis, in cespitem aggrestis* in [26] (p. 20). In Dillenius [18] (p. 308), it was made a synonym for his *Hypnum subhirsutum, viticulis gracilibus erectis, capsulis teretibus* [18] (p. 307), and [19] (p. 210) established for the latter name a binomial *Neckera viticulosa* Hedw., today *Anomodon viticulosus* (Hedw.) Hook. & Taylor. 

**Identification:** the specimens represent ***Anomodon viticulosus* (Hedw.) Hook. & Taylor**. Its status in the vicinity of Węgorzewo is similar to that of *Neckera pinnata* discussed above.

### 4.10. Volume 5, Page 15

Page 15 holds one specimen with one Lat. name: *Muscus squamosus repens tenuissimis foliis*. This polynomial should be cited after Tournefort [12] (vol. 1, p. 554). Dillenius [18] (p. 453) made it a synonym for his *Lycopodium palustre repens, clava singulari* (p. 452), which is *Lycopodiella inundata* (L.) Holub, not a moss.

**Identification:** the specimen is a true moss, ***Eurhynchium angustirete* (Broth.) T.J.Kop.**, characterised above.

### 4.11. Volume 5, Page 16

Page 16 holds one specimen with two names:

Lat.: *Muscus squamosus palustris candicans mollissimus*. It should be cited after [30] (p. 505) and [12] (vol. 1, p. 554). By Dillenius [18] (p. 242), it was made a synonym for his *Sphagnum palustre molle deflexum, squamis cymbiformibus* [18] (p. 240). Hedwig [19] (p. 27) named the latter binomially *Sphagnum latifolium* Hedw. Today, it is *S. palustre* L.

Lat.: *Muscus palustris terrestri similis Raji Hist.* It should be cited after Ray [39] (p. 122). This polynomial was synonymised by [30] (p. 554) with his *Muscus squamosus palustris candicans, mollissimus*, so it is identical to the previous identification.

**Identification:** the specimens are a mixture of ***Sphagnum* cf. *fallax* (H.Klinggr.) H.Klinggr.** and ***Straminergon stramineum* (Dicks. *ex* Brid.) Hedenäs** (only one shoot in the middle part). *S. fallax* is one of the most common sphagnum species in the flora of Poland, associated with oligotrophic peat bogs and marsh forests, currently rare in the vicinity of Węgorzewo [16]. *S. stramineum* is a common species in Poland, growing in peat bogs and marshy forests, but recently not seen in the vicinity of Węgorzewo.

### 4.12. Volume 5, Page 17

Page 17 holds two specimens. The top specimen has one Lat. name: *Muscus squamosus palustris capitulis rufescentibus. Ei prioris varietas.* The polynomial stands without citation. It was first published only in [20] (p. 49), where we also find a remark that it was a variety of *Muscus squamosus palustris candicans mollissimus*. A similar Latin remark is placed in the herbarium: *ei prioris varietas* (i.e., “a variety of the former”), i.e., of the specimen on p. 16. The polynomial *Muscus squamosus palustris capitulis rufescentibus* is found with citation from [20] in [18] (p. 244), where it became a synonym for the Dillenian name *Sphagnum palustre molle deflexum, squamis capillaceis* (p. 243)—more precisely, of its red-tinted variety (*varietas rubens*). By [19] (p. 28), it was named binomially *Sphagnum capillifolium* (Ehrh.) Hedw. 

**Identification:** the specimens represent ***Sphagnum magellanicum* Brid.** (left side) and ***Sphagnum* cf. *rubellum* Wilson** [= *S. capillifolium* (Ehrh.) Hedw. subsp. *rubellum* (Wilson) M.O.Hill] (right side of the page). *S. magellanicum* is a species associated with oligotrophic peat bogs and marsh forests, currently rare in the vicinity of Węgorzewo [16]. *S. rubellum* was found in the vicinity of Węgorzewo in the first half of the 20th century [40]; in recent years, it has not been observed.

The bottom specimen has one Lat. name: *Muscus squamosus capillaceus minimus capitulo longo erecto*. This polynomial is absent in [11,20], i.e., it is designated in the studied herbarium only. 

**Identification:** the bottom specimen represents a mixture of ***Amblystegium serpens* (Hedw.) Schimp.** (mainly right side) and ***Pylaisia polyantha* (Hedw.) Schimp.** (mainly left side). *A. serpens* is one of the most common Polish mosses, growing in various habitats, such as soil, tree bark, and old walls, and, so far, it is common in the vicinity of Węgorzewo [15,16,17]. *P. polyantha* grows mainly on tree bark and less often on old walls and rocks, and it is also a frequent species in Węgorzewo today [15]. The Latin words *capitulo longo erecto* describe well the shape and position of the capsules of *P. polyantha*, so this polynomial must have been intended and designated for this species. 

### 4.13. Volume 5, Page 18

Page 18 holds one specimen with two names.

Lat.: *Muscus squamosus veluti repens spicatus in aquis nascens Tourn*. The words should be reordered as *Muscus squamosus repens, veluti spicatus*, and this version should be cited after Tournefort [41] (p. 554). This author provided older names as synonyms: *Muscus ramosus, repens, spicatus* [27] (p. 361), *Muscus ramosus repens velut spicatus* [42] (p. 351), [35] (p. 625). Out of these polynomials, in [18] (p. 314), only the following can be found: *Muscus ramosus repens C. B. velut spicatus Raj. Hist. I p. 114.* Dillenius made it a synonym for his *Hypnum dendroides sericeum, setis et capsulis longioribus erectis* [18] (p. 313). Meanwhile, [19] (pp. 228–229) named it *Leskea dendroides* Hedw. Today, it is *Climacium dendroides* (Hedw.) F. Weber & D. Mohr. The words *in aquis nascens* (“born in waters”), standing by the original name on the herbarium sheet, are an addition about the habitat, not part of the polynomial of this species. 

Lat.: *Muscus aquaticus ramosus repens veluti spicatus C. B. Prodrom. ad arborum radices*. This polynomial should be cited after Bauhin [42] (p. 151), where we find it as *Muscus ramosus repens velut spicatus ad arborum radices*, i.e., not *aquaticus*. If we believe Tournefort [12] (p. 554), this polynomial was a synonym for *Muscus ramosus repens spicatus* by Bauhin [27] (p. 361), and for *Muscus ramosus repens velut spicatus* by Tournefort [12] (p. 554). These Bauhin and Tournefort polynomials were synonymised by Dillenius [18] (p. 314) with his *Hypnum dendroides sericeum, setis et capsulis longioribus erectis* [18] (p. 313), so it is *C. dendroides*, as above.

**Identification:** the specimens represent ***Climacium dendroides* (Hedw.) F.Weber & D.Mohr var. *fluitans* Huebener**. The occurrence of this species in the vicinity of Węgorzewo was described above.

### 4.14. Volume 5, Page 19

Page 19 holds one specimen with one Lat. name: *Muscus squamosus foliis acutissimis in aquis nascens*. This polynomial should be cited after Tournefort [12] (p. 554). In Dillenius [18] (p. 522), it was made a synonym for his *Fontinalis triangularis major complicata, e foliorum alis capsulifera* [18] (p. 254). Hedwig cited this polynomial as *Fontinalis foliis triangularibus maioribus complicatis, e foliorum alis capsulifera* and, in his work, it is a synonym for *Fontinalis antipyretica* Hedw. [19] (p. 298).

**Identification:** the specimen is ***Fontinalis antipyretica* Hedw.**, a common aquatic species in Poland, but recently it has been observed rarely near Węgorzewo. 

### 4.15. Volume 5, Page 20

Page 20 holds one specimen with one Lat. name: *Muscus aquaticus tenuissimis foliis cauliculis adhaerentibus Loes*. This polynomial should be cited after Loesel [34] (p. 51) and [31] (p. 173). It became a synonym for *Hypnum erectumaut fluitans, foliis oblongis perangustis acutis* in Dillenius [18] (p. 300). Meanwhile, [19] (p. 296) named it *Hypnum fluitans* Hedw. The accepted binomial is *Warnstorfia fluitans* (Hedw.) Loeske. 

**Identification:** the specimen is ***Drepanocladus* cf. *aduncus*** (Hedw.) Warnst., a moss common in Poland, occurring in water and wet habitats, still present in the bryoflora of Węgorzewo [15,17]. 

### 4.16. Volume 5, Page 22

Page 22 holds one specimen with three names:

Lat.: *Muscus Polygoni folio.* This polynomial should be cited after [30] (p. 504) or [12] (p. 555). Later, Dillenius [18] (p. 411) made it a synonym for his *Bryum dendroides polycephalon, Phyllitidis folio undulato pellucido, capsulis ovatis pendulis* [18] (p. 410). It was named *Mnium undulatum* Hedw. [19] (p. 195), today *Plagiomnium undulatum* (Hedw.) T.J.Kop.

Lat.: *Muscus ramosus erectus oblongifolius Loeselii*. This polynomial comes from Loesel’s works: [34] (p. 49) and [31] (p. 168). It was made a synonym for *Bryum dendeoides polycephalon, Phyllitidis folio undulato pellucido, capsulis ovatis pendulis* by Dillenius [18] (p. 410), so it is *P. undulatum* as above. 

Lat.: *Muscus ad Polytrichoiden accedens arbusculam referens foliis oblongis*. It is originally declensed as *Muscus ad Polytrichoidem accedens, arbusculam referens, foliis longis* in [37] (p. 36) and rewritten as “…*Polytrichodem*…” in Dillenius [18] (p. 411). It is there one of the synonyms of the abovementioned species, *P. undulatum*. 

**Identification:** the specimen represents ***Plagiomnium undulatum* (Hedw.) T.J.Kop.**, a species common in Poland, still growing in wet forests and shrubs in the vicinity of Węgorzewo [15,16]. 

### 4.17. Volume 5, Page 23

Page 23 holds one specimen with two names:

Lat.: *Muscus palustris foliis subrotundis Tourn*. This polynomial was published by Tournefort [12] (p. 555). In Dillenius [18] (p. 414), it is made a synonym for *Bryum pendulum, foliis variis pellucidis, capsulis ovatis* [18] (p. 413). It was named by [19] (p. 192) *Mnium cuspidatum* Hedw., today *Plagiomnium cuspidatum* (Hedw.) T.J.Kop. 

Lat.: *Muscus uvida amans, foliis subrotundis expansis Loes.* This polynomial comes from [34] (p. 49) and [31] (p. 168). It was listed by Dillenius [18] (p. 418) as a synonym for his *Bryum pendulum, Serpilli folio rotundiore pellucido, capsulis ovatis* [18] (p. 416). In [19] (p. 194), this Dillenian name was made a synonym for *Mnium punctatum* Hedw.; today, it is *Rhizomnium punctatum* (Hedw.) T.J.Kop. 

**Identification:** the specimen represents ***Plagiomnium elatum* (Bruch & Schimp.) T.J.Kop.**, a moss associated with peat bogs and wet forests, growing so far in the vicinity of Węgorzewo [15,16].

### 4.18. Volume 5, Page 24

Page 24 holds one specimen with two names:

Lat.: *Muscus Nummulariae folio major*. This polynomial should be cited after Tournefort [12] (p. 555) and [29] (p. 99). It was synonymised by Dillenius [18] (p. 483) with his *Lichenastrum asplenii facie pinnis laxioribus* [18] (p. 482). This is *Jungermannia asplenioides* L. [28] (p. 1131), [25] (p. 1597). 

Lat.: *Muscus bifolius procumbens foliis subrotundis Loes*. It should be cited after [31] (p. 167). By Dillenius [18] (p. 483), it was made a synonym for *Lichenastrum asplenii facie pinnis laxioribus* [18] (p. 482). This is *Jungermannia asplenioides* L. (as above).

**Identification:** the specimens are ***Plagiochila* cf. *asplenioides* (L.) Dumort.** This liverwort grows most often in marshy forests and on the banks of streams. Currently, it is a rare species in the vicinity of Węgorzewo [16]. 

### 4.19. Volume 5, Page 25

Page 25 holds one specimen with two names:

Lat.: *Muscus pennatus vulgaris major*. The correct word order is *Muscus vulgaris pennatus major*, and it is a citation of (p. 360). It was made a synonym of *Hypnum pennatum undulatim crispum setis et capsulis brevibus* [18] (p. 283). Having replaced the adverb *undulatim* with the adjective *undulatum*, Hedwig [19] (p. 206) made it a synonym of his *Neckera crispa* Hedw. 

Germ.: *Wald-Feder-Moß,* i.e., “a forest feather moss”. 

**Identification:** the specimens represent ***Ptilium crista-castrensis* (Hedw.) De Not.**, a forest moss, growing mainly on soil and rarely on rotting wood. Currently, it is rare in the vicinity of Węgorzewo [16].

### 4.20. Volume 5, Page 26

Page 26 holds two specimens, each with one name. The top specimen is named in Lat. *Muscus vulgaris pennatus minor*. This polynomial, established in [27] (p. 360), was only discussed by [18] (p. 174) as a possible variety of *Muscus pennatus vulgaris major*. Due to its sparse description in Bauhin’s works, it has not been recognised by subsequent botanists. 

**Identification:** this specimen is again ***Ptilium crista-castrensis* (Hedw.) De Not.**, described above.

The bottom specimen is named in Lat. *Muscus pennatus tectis vetustis insidens*. This polynomial should be cited after [31] (p. 167). In [18] (p. 274), it is renamed as *Muscus pennatus tectis vetustis innascens* and placed among the synonyms of *Hypnum pennatum undulatim crispum, setis et capsulis brevibus* [18] (p. 283), so it is *Neckera crispa* Hedw. as on p. 25 of the herbarium. Latin *tectis vetustis insidens*—“setting on old roofs”, *innascens*—“being born” on them. 

**Identification:** this specimen is ***Dicranum scoparium* Hedw.** The occurrence of this species near Węgorzewo was presented above.

### 4.21. Volume 5, Page 27

Page 27 holds one specimen with one Lat. name: *Muscus pennatus major cauliculis ramosis in summitate veluti spicatus Loes.* This polynomial should be originally spelled …*velut*… and cited after [31] (p. 167 and Fig. 43). In Dillenius [18] (p. 285), it is a synonym for his *Hypnum filicinum, cristam castrensem repraesentans* [18] (p. 284). In [19] (p. 287), this polynomial became a synonym for *Hypnum crista-castrensis* Hedw., today *Ptilium crista-castrensis* (Hedw.) De Not. 

**Identification:** the specimens represent ***Hylocomiadelphus triquetrus* (Hedw.) Ochyra & Stebel** [= *Rhytidiadelphus triquetrus* (Hedw.) Warnst.]. It is a large forest moss, occurring mainly on soil and rarely on rotting wood. At present, it is infrequent in the vicinity of Węgorzewo [15,16].

### 4.22. Volume 5, Page 28

Page 28 holds one specimen with one Lat. name: *Muscus pennatus minor cauliculis ramosis in summitate veluti spicatus Loes.* This polynomial comes from [31] (p. 167). In Dillenius [18] (p. 284), renamed as …*velut*…, it became a synonym of his *Hypnum repens filicinum crispum* [18] (p. 282). It was also added in the third edition of [43] (p. 85). Withering treated it as a synonym for his *Hypnum filicinum* [44] (p. 684), and [19] named it binomially *H. filicinum* Hedw. [19] (p. 285). The latter is today *Cratoneuron filicinum* (Hedw.) Spruce.

**Identification:** this is ***Cirriphyllum piliferum* (Hedw.) Grout**, the species discussed above (vol. 5, p. 6).

### 4.23. Volume 5, Page 29

Page 29 holds one specimen with one Lat. name: *Muscus filicinus major Tourn.* It should be cited after Bauhin [27] (p. 360) and was only repeated in Tournefort [12] (p. 556). It has never been used by newer authors and is missing in both [18,19]. It has been recently proposed to be a name of *Thuidium delicatulum* (Hedw.) Schimp. (=*Hypnum delicatulum* Hedw.) [22]; for a discussion, see therein. 

**Identification:** the specimens represent ***Hylocomium splendens* (Hedw.) Schimp.**, a moss mainly occurring on the forest floor in coniferous forests, frequent in the vicinity of Węgorzewo [15,16].

### 4.24. Volume 5, Page 30

Page 30 holds one specimen with two Lat. names: *Muscus filicinus minor floridus C. B. prodr. Capitula Adianthi autumno aliquando produist*. It should be cited after [42] (p. 151). The identification of this polynomial name, as we published in [22], led to as many as three possible modern taxa: either *Thuidium tamariscinum* (Hedw.) Schimp., *Hylocomium splendens* (Hedw.) Schimp., or *Kindbergia praelonga* (Hedw.) Ochyra. 

**Identification:** the specimens belong to ***Thuidium* cf. *assimile* (Mitt.) A.Jaeger**, a moss occurring on the edges of forests, in grasslands, and on roadside slopes, still growing in the vicinity of Węgorzewo [15,16].

### 4.25. Volume 5, Page 71

Page 71 holds one specimen with two polynomials:

Lat.: *Corallina Cupressiformis ramosa*. This polynomial does not occur in sources and is a source of confusion. *Corallina* has been applied to plant-like, coral-shaped organisms since at least the 16th century. Tabernaemontanus [36] (p. 810) imaged certain lichen and named it *muscus corallinus sive corallina montana*; in other cases, he applied the name *corallina* to marine algae (pp. 811, 813) or true corals (pp. 1122, 1123). Tournefort [12] (pp. 570–572) used the noun *corallina* interchangeably with *muscus* and *fucus* in the polynomials of marine organisms. The reason for the application of this polynomial for a moss is the adjective *cupressiformis*; compare below. 

Lat.: *Muscus cupressiformis ramosus Loes.* This polynomial is present in [31] (p. 168), authorised by himself and imaged on plate 48. In [18] (p. 310), it is authorised as Bauhin [27] (p. 361, “n. 9”), and Dillenius [18] (p. 309) made it a synonym for his *Hypnum cupressiforme vulgare, foliis obtusis*. The latter polynomial became in [19] (p. 255) a synonym for *Hypnum purum* Hedw., today *Pseudoscleropodium purum* (Hedw.) M. Fleisch. ex Broth.

Germ.: *Aestiger Cÿpressen-Moß*. This name means “gnarled cypress-moss” and is published in [31] (p. 168). 

**Identification.** The specimen represents ***Pleurozium schreberi* (Willd. *ex* Brid.) Mitt.**, one of the commonest forest mosses in Poland; at present, it is frequent in the vicinity of Węgorzewo [15,16].

### 4.26. Volume 4, Page 252

Page 252 holds one specimen with four names. 

Lat.: *Lichen petraeus latifolius, s: Hepatica fontana. Loes*. This polynomial is cited after [27] (p. 362) and repeated in Mentzel [45] (p. 218), [34] (p. 42) and [31] (p. 140). It was made a synonym for *Marchantia polymorpha* L. [28] (p. 1137). 

Germ.: *Stein-flechten Moß-flechten*, i.e., “stone-lichen, moss-lichen”. 

Germ.: *Grünnen-Leber-Kraut*, i.e., “a green liver-herb”. 

Pol.: *Wątrobie ziele. Liszajec*, i.e., “a liver herb, a lichen”. The Pol. name “*wąrtobie źiele*” was published in [20] (p. 41).

**Figure 2:**
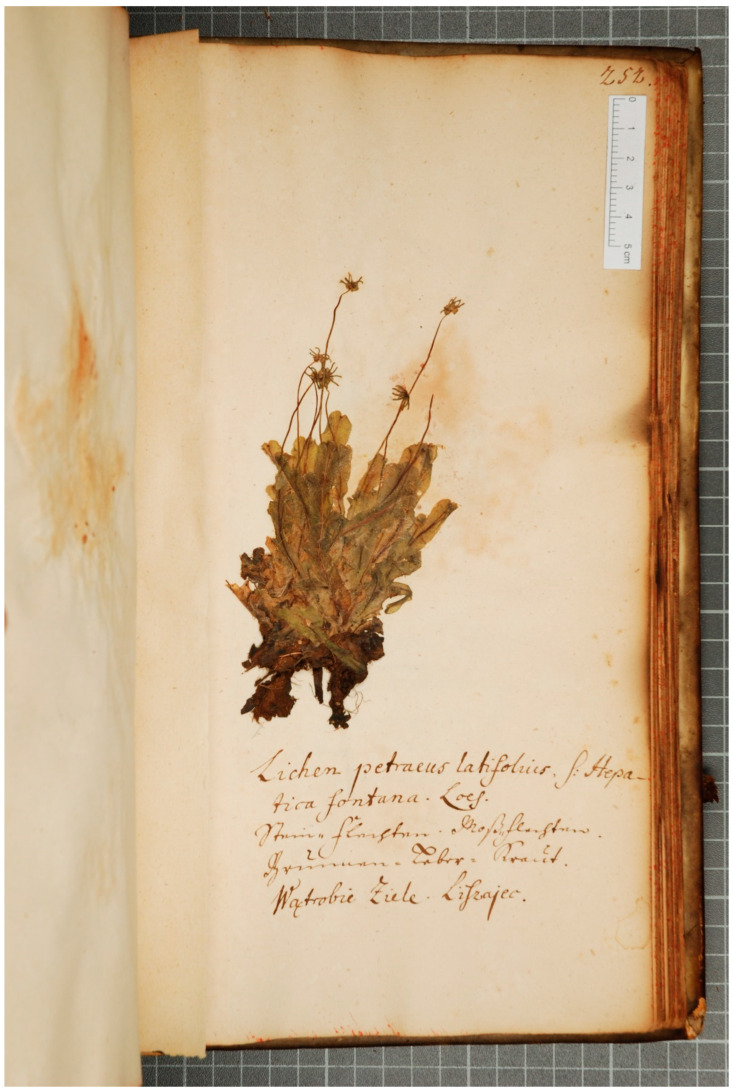
*Marchantia polymorpha* L. subsp. *polymorpha* in the WA copy of Boretius’ herbarium from 1717. Photograph by M. Graniszewska. Transcription: *Lichen petraeus latifolius, s: Hepatica fontana. Loes. Stein-flechten Moß-flechten. Grünnen-Leber-Kraut. Wątrobie ziele. Liszajec* (see Section 4.26 for translation and interpretation).

**Identification:** the specimens stand for female individuals of ***Marchantia polymorpha* L. subsp. *polymorpha*** [=*M. aquatica* (Nees) Burgeff]. This species is confined to wet habitats, presently rare near Węgorzewo [16]. See Figure 2. 

### 4.27. Volume 4, Page 253

Page 253 holds one specimen with four names. 

Lat.: *Lichen petraeus stellatus. C. B.* This name is cited after [27] (p. 362). It was made a synonym for *Marchantia polymorpha* L. [28] (p. 1137). It represents specimens with archegoniophores.

Lat.: *Hepatica Tabern: secunda.* It should be cited after [36] (p. 815). It was considered as the variety β (now discarded) of *M. polymorpha* L. [28] (p. 1137). 

Germ.: *Kleiner gestirnte Stern-flechten oder Leber-Kraut*, i.e., “smaller starred star-lichen or liver herb”. A simpler name, *gestirnt Leberkraut* (“a starred liver herb”), was published in [20] (p. 41).

Pol.: *Wątrobiec* is a neologism stemming from Pol. *wątroba*—”the liver”.

**Identification:** The specimens are female individuals of ***Marchantia polymorpha* L. subsp. *ruderalis* Bischl. & Boissel.-Dub.** [=*M. latifolia* Gray]. It is one of the commonest liverworts in Poland, still present in the bryoflora of Węgorzewo [15].

### 4.28. Volume 4, Page 254

Page 254 holds one specimen with three names. 

Lat.: *Lichen petraeus umbellatus.* This is a polynomial by Bauhin [27] (p. 362). It was made a synonym of the variety γ (now discarded) of *Marchantia polymorpha* L. [28] (p. 1137).

Lat.: *Hepatica tertia Tabern.* This is a polynomial by [36] (p. 492). It was repeated (as “*Hepatica 3. Tab.*”) as a synonym of *Lichen petraeus umbellatus* by [46] (p. 42).

Germ.: *Leber-Kraut mit runden breiten Köpfchen.*, i.e., “a liver herb with round broad heads”. This German name was published by [20] (p. 41) and repeated in [46] (p. 42). Both the Lat. adjective *umbellatus* and this German name describe specimens with antheridiophores.

**Identification:** the specimens are male individuals of ***Marchantia polymorpha* L. subsp. *ruderalis* Bischl. & Boissel.-Dub.** (compare above). 

## 5. Discussion

### 5.1. List of Identified Species

Table 1 lists the bryophyte species found in Boretius’ herbarium that were identified correctly by him, as well as their medicinal importance in his time.

Out of the nine species gathered in Table 1 as identified correctly (i.e., which Boretius knew well, and excluding his attempt to designate *P. polyantha*), six bryophytes are now common or frequent around the place in which he lived. Moreover, two of them were of medicinal importance already in his time: *Marchantia polymorpha*, which is seen on as many as three sheets in various forms of both sexes, and *Polytrichum commune*, which is on sheet 5.2 and also on 5.3 (Table 2).

Table 2 shows the misidentified species. Boretius’ polynomials can be resolved today as 21–23 binomials. However, our independent taxonomic check yielded 25 real (but different) taxa and revealed the essential difficulties that Boretius had encountered in his practice: some of these taxa are represented by more than one specimen, while four gatherings are clusters formed by more than a single species. 

### 5.2. Boretius as a Bryologist

**Taxonomy.** Bryophyte species were generally misidentified. The names written on the herbarium sheets are correct for 10 of out of real 28 species. Of these, both species of liverwort are correctly identified.

**Boretius’ sources.** Boretius made extensive use of the botanical literature of his time, or at least knew many authors of bryophyte polynomials, including the oldest local botanists: [34,45]. Both these writers, Mentzel and Loesel, should be considered as the pioneers of the local Prussian flora. He also mentioned a Dutch author, Hermann Boerhaave, who was generally recognised as an influential and skilful physician rather than a *materia medica* writer.

**Designation of polynomials.** In the herbarium studied, we divulgated two polynomials of mosses used for the first time. (1) In vol. 5 p. 17 (top), Boretius attempted to designate *Sphagnum capillifolium* (Ehrh.) Hedw. In fact, there are two different peat mosses here (also *S. magellanicum* Brid.) and it is not clear which one Boretius had in mind. (2) At the bottom of the same page, he effectively designated *Pylaisia polyantha* (Hedw.) Schimp. as *Muscus squamosus capillaceus minimus capitulo longo erecto*. This name was repeated in [20] (p. 49) without authorship.

**Boretius’ collection as voucher material for botanical publications of the time.** All inscriptions present on the investigated herbarium sheets are written by one hand [6]. The youngest polynomial used is *Muscus squamosus palustris capitulis rufescentibus*, for which the only published source is a list from 1726 [20], where it is established in print. Helwing intended this book [20] as a supplement to the flora of East Prussia [11] (issued in 1712). Thus, Boretius’ herbarium may have been expanded or completed after 1717, also as a reference for this supplement. A similar study of the flowering plants in the herbarium should be undertaken to support this thesis.

### 5.3. Folk Medicine and Vernacular Names of Bryophytes

Some Polish and German vernacular phytonyms were written on herbarium sheets. We can consider their existence as proof of their ethnobotanical knowledge and possible use. There are 13 German names and 4 Polish names. The German *Groß gülden Wieder-Todt* = *Frauen oder Venus-Haar* and Polish *Matki Bożey włoski* are of medicinal origin. The name Venus’ hair is generally applied to the medicinal fern *Adiantum capillus-veneris* L., native to the Mediterranean region. In Central Europe, this name was transposed to some representatives of the genus *Polytrichum* Hedw. These herbs, generally called, in pharmacy, “capillary herbs” (Lat. *herbae capillares*, e.g., in [47]), were believed to be a good remedy for hair and scalp disorders, thanks to the fact that the dried herb of *A. capillus-veneris* is conspicuous for its hair-thin petioles. According to the doctrine of signatures, this property was noticed in the herb of Central European mosses of the genus *Polytrichum*, which is even better represented here by the presence of thin, long, pale, and glittering trichomes on the calyptra, which together resemble a blonde female hairstyle. In Europe, *Polytrichum* was first reported as a moss of medicinal value as early as in 1549 [21]). 

Another vernacular German name, *Klein Wieder-Todt oder Venus-Haar*, stands next to *Funaria hygrometrica* Hedw., a medicinal moss used before 1600 [21].

Vernacular names for liverworts associate them with the liver: Germ. *Leber-Kraut*, *Grünnen-Leber-Kraut*, *Kleiner gestirnte Stern-flechten Leber-Kraut*; Pol. *Wątrobie ziele*, and *Wątrobiec*. Other names, Germ. *Stein-flechten Moß-flechten* and Pol. *liszajec*, refer to the skin conditions called, in Latin, *lichen planus* (and *impetigo contagiosa*). It is generally accepted that, according to the doctrine of signatures, thalloid liverworts were used in ancient medicine in two ways: as liver remedies (thanks to the resemblance of their gametophytes to liver lobes) and in skin diseases called “lichen” (due to the shape and size of the “spots” on the patient’s skin).

Boretius misidentified other mosses that were considered medicinal in his time and mentioned by other writers as articles of material medica. They were *Funaria hygrometrica*, *Hylocomiadelphus triquetrus*, and *Brachythecium rutabulum*. This omission or error may prove their insignificant role in the therapy of the time.

There are other observations about Boretius’ expertise in bryology that are important for our view of bryophyte-based herbalism:

- He sometimes used several polynomials that were not taxonomically equivalent, e.g., in 5.23 (=vol. 5, p. 23, all examples are collected in Table 2).
- He identified three species mixed in a cluster as one (5.5).
- He misidentified true mosses as representatives of the genera of lycopods *Lycopodiella inundata* and *Selaginella denticulata* (5.9, 5.15).
- He encountered objective difficulties in distinguishing between morphologically similar species (see 5.71; 5.17 top; 5.17 bottom).
- He filled as many as three sheets of his herbarium with various forms of *Marchantia polymorpha*. Together with the rich folk nomenclature, this proves the good knowledge of this liverwort and suggests its popularity as a medicinal plant in 1717.

Most of the mosses present in the herbarium and listed in Table 2 had little chance of being promoted as medicinal plants, either because of their rarity in the environment or because of identification problems. Thus, even common species (such as *Amblystegium serpens*, *Ceratodon purpureus*, *Hylocomium splendens*, *Pohlia nutans*) could not objectively have been learned by doctors, pharmacists, botanists, herbalists, or patients harvesting herbal material for themselves. It must be remembered that the confirmation of taxonomic identity was the most desirable (and usually the only available) method of proving the identity, authenticity, and quality of medicinal plant materials in times when any assessment of chemical composition was far beyond the remit of quantitative and qualitative chemical analysis.

Boretius created his herbarium at a time when the microscope was not yet in use for the study of bryophytes. Dillenius [18], in the preface to his outstanding *Historia Muscorum* (on both the taxonomy and nomenclature of bryophytes), stated (on p. xv) that, at least in the case of the former genus *Hypnum*, there were structural details “*…quae oculis nostris patent vel nudis, vel lente vitrea mediocri armatis*”—“which are accessible to our eyes either naked or through a glass lens of medium power”. We queried his book for the Latin words *microscopium* and lens (which both refer to “a microscope, a loupe, a magnifying glass”) and for their declensed forms (genitive, accusative, and ablative case), and it was found that he mentioned microscopy at least 22 times in his text. Finally, Hedwig (1730–1799) was the first bryologist to use magnifications of up to 290× [19] as a standard in his research, which resulted in his fundamental illustrated work *Species Muscorum*… Thus, Boretius, who died in 1738, did not have sufficient tools to distinguish or harvest the herbal material from the world of bryophytes according to the method and requirements of scientific pharmacy at that time.

## 6. Conclusions

Among the specimens of bryophytes found in Boretius’ herbarium, two species and one subspecies of liverwort and 27 species and one variety of moss were identified. Most of them are common species that still grow in the vicinity of Węgorzewo, so Boretius gathered his bryophytes locally.

Newly discovered historical sources show 1717 as the date of origin of Boretius’ herbarium. This date justifies the poor taxonomic knowledge of bryophytes and proves their marginal role as a source of medicinal herbal material at the time that the collection was created. Thus, the motivation for creating this herbarium in 1717 was the plant taxonomy or floristics of bryophytes, rather than the economic/medicinal use of them.

If the 1717 herbarium proved to be unreliable support for the taxonomy of bryophytes, the botanical–medical texts of the time, which mention bryophytes as medicinal plants, must be even less reliable (as they are not even accompanied by voucher specimens). Even conspicuous mosses such as *Polytrichum* are subject to error in this herbarium. Even when Helwing identified the specimens for Boretius, the latter repeatedly made mistakes when mounting them on the herbarium sheets and combined them with incorrect names. We should therefore doubt the certainty of the recognition of bryophyte species as medicinal plants in the past and consider the taxonomic identity of the former moss-derived herbal materials as ambiguous.

Polish and German vernacular names appeared in Boretius’ herbarium next to the species best known as medicinal from the 16–18th-century literature. He correctly recognised only two medicinal bryophyte species (*Polytrichum commune* and *Marchantia polymorpha*), and the folk names of both are related to old folk medicine or pharmacy.

An increase in the number of medicinal bryophyte species did not occur until the second half of the 18th century. This can be explained by the influence of the taxonomic–nomenclatural work of Dillenius [18], who used microscopic features to identify species. Taxonomic knowledge at the time of Helwing and Boretius (early 18th century) was limited to two to three species of bryophytes already known in antiquity or introduced for therapeutic purposes in the early Renaissance (as *Marchantia*).

## Figures and Tables

**Table 1:** Bryophytes included in Boretius’ herbarium that are correctly identified therein, together with their historical medicinal use and contemporary occurrence in the local flora of Węgorzewo.

Volume, Page, Placement	Confirmed Taxonomical Identity	Medicinal Importance in 1717 [21]	Frequency in the Local Flora Today
4.252–4.254	*Marchantia polymorpha*	yes, since antiquity	common
5.2	*Polytrichum commune*	yes, since 1549	frequent
5.3	*Dicranum scoparium*	no	common
5.5	*Rhodobryum roseum*	no	rare
5.13	*Anomodon viticulosus*	no	very rare
5.17 bottom	species designation of *Pylaisia polyantha* (but compare 5.17 in Table 2)	no	common
5.18	*Climacium dendroides*	no	frequent
5.19	*Fontinalis antipyretica*	later, since 1755	very rare
5.22	*Plagiomnium undulatum*	no	frequent
5.24	*Plagiochila asplenioides*	no	very rare

**Table 2:** Bryophytes included in Boretius’ herbarium that are misidentified in it, together with the historical medicinal use of the species misidentified by Boretius, and with the contemporary occurrence in the local flora of Węgorzewo of the real taxon represented in the herbarium.

Volume, Page, Placement	Taxonomical Identification by Boretius (Boretius’ Polynomial Would Lead to…)	Medicinal Importance of Boretius’ Species in 1717 [21]	Correct Taxonomical Identification of the Species	Frequency in the Local Flora Today
5.1	*Polytrichum juniperinum*	later, in the 20th century	*Polytrichum commune* with admixture of *Polytrichum* cf. *strictum* (=*P. juniperinum* subsp. *strictum*)	frequent
5.4	*Funaria hygrometrica*	yes, since 1583 or 1600	*Ceratodon purpureus* and *Pohlia nutans*	both common
5.6 top	*Hylocomiadelphus triquetrus*	possible, since 1651	*Cirriphyllum piliferum*	frequent
5.6 bottom	*Brachythecium rutabulum* and *Brachytheciastrum velutinum*	yes (*B. rutabulum*), since 1651	*Eurhynchium angustirete*	frequent
5.8	*Rhytidiadelphus loreus and Plagiothecium undulatum*	no	*Climacium dendroides*	frequent
5.9 top	*Selaginella helvetica*	no	*Neckera pennata*	very rare
5.9 bottom	*Selaginella denticulata*	no	*Neckera pennata*	very rare
5.15	*Lycopodiella inundata*	no	*Eurhynchium angustirete*	frequent
5.16 top and bottom	*Sphagnum palustre*	later, since 1883	*Sphagnum* cf. *fallax*	rare
5.17 top	*Sphagnum capillifolium*	later, since 1884	*Sphagnum magellanicum* and *S.* cf. *rubellum*	*S. mag.* rare, *S. rub.* not observed
5.17 bottom			*Amblystegium serpens* (admixed to *Pylaisia polyantha*)	both common
5.20	*Warnstorfia fluitans*	no	*Drepanocladus* cf. *aduncus*	frequent
5.23	*Plagiomnium cuspidatum and Rhizomnium punctatum*	no/no	*Plagiomnium elatum*	rare
5.25	*Neckera crispa*	no	*Ptilium crista-castrensis*	rare
5.26 top	?		*Ptilium crista-castrensis*	rare
5.26 bottom	*Neckera crispa*	no	*Dicranum* cf. *scoparium*	common
5.27	*Ptilium crista-castrensis*	later, since 1787	*Hylocomiadelphus triquetrus*	frequent
5.28	*Cratoneuron filicinum*	no	*Cirriphyllum piliferum*	frequent
5.29	*Thuidium delicatulum*	no	*Hylocomium splendens*	frequent
5.30	*Thuidium tamariscinum or Hylocomium splendens or Kindbergia praelonga*	yes (only *Th. tamariscinum*), since 1656	*Thuidium* cf. *assimile*	frequent
5.71	*Pseudoscleropodium purum*	no	*Pleurozium schreberi*	common

## Data Availability

The studied collection is housed at the WA Herbarium, Faculty of Biology, University of Warsaw, ul. Żwirki i Wigury 101, 02-089 Warszawa, Poland.

## Abbreviations

cf.—probably; fig.—figure; Germ.—German; Lat.—Latin; p.—page; Pol.—Polish; subsp.—subspecies; var.—variety; vol.—volume.
